# Genome-scale determination of 5´ and 3´ boundaries of RNA transcripts in *Streptomyces* genomes

**DOI:** 10.1038/s41597-020-00775-w

**Published:** 2020-12-15

**Authors:** Yongjae Lee, Namil Lee, Soonkyu Hwang, Woori Kim, Yujin Jeong, Suhyung Cho, Bernhard O. Palsson, Byung-Kwan Cho

**Affiliations:** 1 Department of Biological Sciences and KI for the BioCentury, Korea Advanced Institute of Science and Technology, Daejeon, 34141 Republic of Korea; 2 Department of Bioengineering, University of California San Diego, La Jolla, CA 92093 USA; 3 Department of Pediatrics, University of California San Diego, La Jolla, CA 92093 USA; 4 Novo Nordisk Foundation Center for Biosustainability, Technical University of Denmark, Lyngby, 2800 Denmark; 5 Intelligent Synthetic Biology Center, Daejeon, 34141 Republic of Korea

**Keywords:** Next-generation sequencing, Bacterial transcription

## Abstract

*Streptomyces* species are gram-positive bacteria with GC-rich linear genomes and they serve as dominant reservoirs for producing clinically and industrially important secondary metabolites. Genome mining of *Streptomyces* revealed that each *Streptomyces* species typically encodes 20–50 secondary metabolite biosynthetic gene clusters (smBGCs), emphasizing their potential for novel compound discovery. Unfortunately, most of smBGCs are uncharacterized in terms of their products and regulation since they are silent under laboratory culture conditions. To translate the genomic potential of *Streptomyces* to practical applications, it is essential to understand the complex regulation of smBGC expression and to identify the underlying regulatory elements. To progress towards these goals, we applied two Next-Generation Sequencing methods, dRNA-Seq and Term-Seq, to industrially relevant *Streptomyces* species to reveal the 5´ and 3´ boundaries of RNA transcripts on a genome scale. This data provides a fundamental resource to aid our understanding of *Streptomyces*’ regulation of smBGC expression and to enhance their potential for secondary metabolite synthesis.

## Abstract (summary)

**Table:** 

Measurement(s)	5´-ends of transcripts • 3´-ends of transcripts • RNA • TSS • transcription_termination_signal
Technology Type(s)	dRNA-Seq • Term-Seq • RNA sequencing
Factor Type(s)	Streptomyces growth phase
Sample Characteristic - Organism	Streptomyces avermitilis • Streptomyces clavuligerus • Streptomyces coelicolor • Streptomyces griseus • Streptomyces lividans • Streptomyces tsukubensis • Streptomyces venezuelae

Machine-accessible metadata file describing the reported data: 10.6084/m9.figshare.13259393

## Background & Summary

*Streptomyces* species are gram-positive filamentous bacteria and hold a great importance for their ability to produce a wide range of clinically or industrially important secondary metabolites^1,2^. During the middle 20th century, the number of available antibiotics rapidly increased and especially, more than 70% of the antibiotics from bacteria were discovered from *Streptomyces* species, emphasizing their importance as the dominant source of antimicrobial compounds^3^. However, the discovery of novel antibiotics rapidly decreased during the latter part of 20th century as research progress with *Streptomyces* species declined as reflected by a decreasing number of novel secondary metabolite discovered^4^. Fortunately, with the emergence of Next-Generation Sequencing (NGS) technique, the genome sequences of many *Streptomyces* species have been collected and increased the potential to produce novel secondary metabolites^5^. Computational prediction revealed that a single *Streptomyces* species typically possesses about 20–50 secondary metabolite biosynthetic gene clusters (smBGCs), and the great number of smBGCs in *Streptomyces* genomes encourages researchers to revisit these organisms to cope with the threat of emerging multi-drug resistant bacteria^6,7^.

**Fig. 1:**
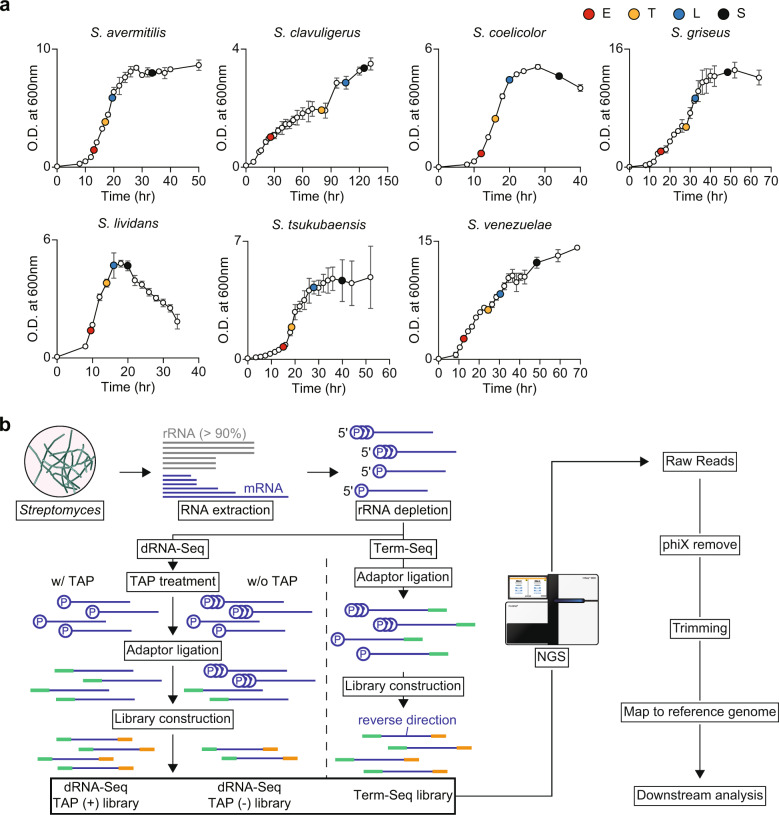
Experimental design and workflow. (**a**) Growth profiles and sampling time points of the seven *Streptomyces* species in R5− media. (**b**) Work flow of dRNA-Seq and Term-Seq. dRNA-Seq and Term-Seq differ in the directions of sequencing adaptor ligation. For dRNA-Seq, two libraries are prepared with or without RNA 5′ polyphosphatase (TAP) to differentiate transcription start sites from the 5′-ends of processed transcripts.

Despite their potential for the production of diverse secondary metabolites, most of the smBGCs have not been characterized in terms of their products and corresponding molecular functions, mainly due to the silent nature of the smBGCs under the laboratory culture conditions^8^. Since most secondary metabolites are not essential for growth and produced to respond to environmental stimuli, such as osmotic pressure or nutrient limitations or inter-species competition, the smBGCs are expected to be under tight and complex regulation^9–11^. To utilize the genomic potential of *Streptomyces*, an understanding of the genetic regulatory mechanisms for activating smBGCs is crucial. Especially, understanding transcriptional regulatory mechanisms is important since the transcription is the first step of gene expression and diverse regulations take place in transcription^12,13^. Here we report NGS datasets describing the differential RNA-Seq (dRNA-Seq) and Term-Seq of seven important *Streptomyces* species to broaden our understanding on transcriptional regulation of *Streptomyces* in genome-scale by providing the transcript boundary information (Fig. 1)^14–17^.

The seven species studied include *Streptomyces avermitilis*, *Streptomyces clavuligerus*, *Streptomyces coelicolor*, *Streptomyces griseus*, *Streptomyces lividans*, *Streptomyces tsukubaensis* and *Streptomyces venezuelae*, widely known for their scientific, clinical and industrial importance. *S. coelicolor* is the most well-known model species and *S. avermitilis*, *S. clavuligerus*, *S. griseus* and *S. tsukubaensis* are known for the ability to produce the anthelmintic agent, avermectin, β-lactamase inhibitor, clavulanic acid, antibiotics, streptomycin, and immunosuppressive agent, FK506, respectively^5,18–21^. *S. lividans* and *S. venezuelae* are majorly used for industrial applications for heterologous expression of proteins and the smBGCs of other *Streptomyces*, since the genetic backgrounds are suitable for heterologous expressions for the two organisms^22,23^. Total RNAs of *Streptomyces* were pooled from four different growth phases, including early-exponential, transition, late-exponential and stationary phases, to cover genes expressed under starvation condition as well as genes involved in primary metabolism at the active growth (Fig. 1a)^24^. dRNA-Seq reveals the transcription start sites (TSSs) of transcripts by differentiating the TSSs from the 5′-ends of processed transcripts. For dRNA-Seq, two libraries are constructed, one from the 5′-ends of unprocessed bacterial primary transcripts and the other from the 5′-ends of processed transcripts. By comparing the two libraries, TSSs can be differentiated from the processed 5′-ends. In contrast, Term-Seq captures the 3′-ends of transcripts, which lead to identification of the genuine transcription termination sites (TTSs) and processed 3′-ends^25^.

From the TSSs determined from dRNA-Seq, the promoter sequences can be identified with the aid of computational motif discovery tools^26^. In addition, TSS information enables to determine 5′-untranslated region (5′-UTR) of each gene in nucleotide resolution, which contains transcriptional or translational regulatory elements, such as the ribosome binding site (RBS), riboswitches and upstream open reading frames^15,27–29^. Likewise, transcriptional terminator sequences and 3′-UTR can be determined from the 3′-end information of transcripts obtained from Term-Seq. With the aid of genome-wide transcriptome and translatome information which can be obtained from RNA-Seq and Ribo-Seq, respectively, the transcriptional and translational effect of each regulatory element, including the promoter sequence, RBS or transcription terminator sequence, can be evaluated. Furthermore, the determined regulatory elements can be utilized for improving the production of secondary metabolites in *Streptomyces* through synthetic biology approaches. The transcript boundary information obtained from dRNA-Seq and Term-Seq will serve as fundamental resources to understand the complex regulatory mechanisms in bacteria and improve the industrial applications.

## Methods

### Strain and culture condition

*S. avermitilis* MA-4680, *S. clavuligerus* ATCC27064, *S. coelicolor* M145, *S. griseus* NBRC13350, *S. lividans* TK24, *S. tsukubaensis* NBRC108819 and *S. venezuelae* ATCC15439 were used in this study. The mycelium of each *Streptomyces* was maintained in 25% glycerol at −80 °C. Cells were cultured in 50 mL R5− media with 8 g glass beads (3 ± 0.3 mm diameter) at 30 °C. The R5− medium consists of 5.73 g TES (pH 7.2), 103 g sucrose, 10 g glucose, 5 g yeast extract, 10.12 g MgCl_2_∙6H_2_O, 0.25 g K_2_SO_4_, 0.1 g casamino acids, 0.08 mg ZnCl_2_, 0.4 mg FeCl_3_∙6H_2_O, 0.02 mg CuCl_2_∙2H_2_O, 0.02 mg MnCl_2_∙4H_2_O, 0.02 mg Na_2_B_4_O_7_∙10H_2_O, and 0.02 mg (NH_4_)_6_Mo_7_O_24_∙4H_2_O in 1 L distilled water. Cell growth was determined by measuring optical density at 600 nm with biological triplicates. The cells were sampled at four different time points according to the growth profile of each strain, which were early-exponential (E), transition (T), late-exponential (L), and stationary (S) phases. The E, T, L, and S time points were 13, 17, 19.5, 33.5 h for *S. avermitilis*, 26, 80, 105.5, 125 h for *S. clavuligerus*, 12, 16, 20, 34 h for *S. coelicolor*, 15.5, 28, 32.5, 48.5 h for *S. griseus*, 9.5, 14, 16, 20 h for *S. lividans*, 13, 19, 23, 31 h for *S. venezuelae*, and 15, 18.5, 28, 48 h for *S. tsukubaensis* after inoculation, respectively (Fig. 1a). For NGS library preparation, cultures for each strain were inoculated in eight flasks as biological octuplicates and cells were harvested from two flasks for each growth phase as biological duplicates.

### RNA extraction

After harvesting, the cells were washed with polysome buffer (20 mM Tris-HCl pH 7.5, 140 mM NaCl, 5 mM MgCl_2_), and resuspended with lysis buffer (0.3 M sodium acetate pH 5.2, 10 mM EDTA, 1% Triton X-100). The cell suspension was frozen with liquid nitrogen, and then physically lysed by grinding using mortar and pestle. The cell lysate was centrifuged at 4 °C for 10 min at 16000 × g and the supernatant was saved and stored at −80 °C until used for RNA extraction. For RNA extraction, the supernatant was mixed with equal volume of phenol:chloroform:isoamyl alcohol = 25:24:1 solution. The mixture was then centrifuged and RNA was extracted from the upper aqueous phase with ethanol precipitation.

For Term-Seq of *S. coelicolor* and *S. griseus*, RNA was extracted by lysing cells with hot phenol. The harvested cells were resuspended with Sol 1 (25 mM Tris-HCl pH 8.0, 10 mM EDTA, 50 mM glucose, 2 mg/mL lysozyme) and incubated at 30 °C for 10 minutes. After incubation, the cells were centrifuged down and the supernatant was discarded. The cell pellet was resuspended with AE-SDS (50 mM sodium acetate pH 5.2, 10 mM EDTA, 1% sodium dodecyl sulfate) and the suspension was mixed with equal volume of phenol:chloroform = 5:1 solution. Cells were lysed by incubating at 65 °C for 5 min and centrifuged. RNA was extracted from the upper aqueous phase with isopropanol precipitation, and genomic DNA aggregate formed upon addition of isopropanol was removed before precipitation.

To remove any DNA contamination, the RNA samples were treated with DNase I (New England Biolabs, Ipswich, MA, USA).

### dRNA-Seq library preparation

The four DNase I treated RNA samples from the four growth phases were mixed equally to obtain one 10 μg RNA mixture and a total of two RNA mixtures were prepared from the eight RNA samples as the biological duplicates for each strain. The rRNA in the RNA mixture was depleted using Ribo-Zero rRNA Removal Kit for Bacteria (Epicentre, Madison, WI, USA). The rRNA-depleted RNA was incubated in 1 × RNA 5′ polyphosphatase (TAP; Epicentre) reaction buffer and 1 U of SUPERase-In (Invitrogen, Carlsbad, CA, USA) at 37 °C for 1 h, with or without TAP for TAP( + ) or TAP(−) libraries, respectively. The reaction was cleaned up with ethanol precipitation and 5 pmol of 5′ RNA adaptor (5′-ACACUCUUUCCCUACACGACGCUCUUCCGAUCU-3′) was ligated to the purified RNA using T4 RNA ligase (Thermo Fisher Scientific, Waltham, MA, USA) by incubating at 37 °C for 90 min in 1 × RNA ligase buffer and 0.1 mg/mL BSA. The ligation product was then purified using Agencourt AMPure XP beads (Beckman Coulter, Brea, CA, USA) according to the manufacturer’s instructions. The purified product was reverse-transcribed with SuperScript III Reverse Transcriptase (Invitrogen) according to the manufacturer’s instructions and purified using Agencourt AMPure XP beads. The purified cDNA was amplified and indexed using Phusion High-Fidelity DNA Polymerase (Thermo Fisher Scientific) for Illumina sequencing. The amplification step was monitored using a CFX96 Real-Time PCR Detection System (Bio-Rad Laboratories, Hercules, CA, USA) and stopped before the PCR reaction was fully saturated. Finally, the amplified library was purified using Agencourt AMPure XP beads.

### Term-seq library preparation

Term-Seq libraries for six species except *S. coelicolor* were prepared as previously described^15,17^. The equal amounts of DNase I-treated RNA from the sampling time points were mixed and used for the input of Term-Seq library construction. The RNA was treated with Ribo-Zero rRNA Removal Kit for Bacteria (Epicentre) to deplete rRNA. The resulting 500~900 ng of rRNA-depleted RNA was mixed with 1 μL of 150 μM amino-blocked DNA adaptor (5′-p-NNAGATCGGAAGAGCGTCGTGT-3′), 2.5 μL of 10 × T4 RNA ligase 1 buffer, 2.5 μL of 10 mM ATP, 2 μL of DMSO, 9.5 μL of 50% PEG8000, and 2.5 μL of T4 RNA ligase 1 (New England BioLabs). The mixture was incubated at 23 °C for 2.5 h and reaction was cleaned-up using Agencourt AMPure XP beads. The adaptor ligated RNA was then fragmented by incubating at 72 °C for 90 seconds in fragmentation buffer (Ambion, Inc, Austin, TX, USA). The fragmentation reaction was cleaned-up using Agencourt AMPure XP beads. The fragmented RNA (8 μL in total) was reverse transcribed with SuperScript III Reverse Transcriptase using 1 μL of 10 μM reverse transcription primer (5′-TCTACACTCTTTCCCTACACGACGCTCTTC-3′) according to the manufacturer’s instructions. The cDNA was then purified with Agencourt AMPure XP beads. Another amino-blocked adaptor with different sequence (5′-p-NNAGATCGGAAGAGCACACGTCTGAACTCCAGTCAC-3′) was ligated to the cDNA with increased incubation time (8 h). The ligation product was purified using Agencourt AMPure XP beads and indexed by PCR with Phusion High-Fidelity DNA Polymerase using forward (5′-AATGATACGGCGACCACCGAGATCTACACTCTTTCCCTACACGACGCTCT-3′) and reverse (5′-CAAGCAGAAGACGGCATACGAGATNNNNNN (6 nt index) GTGACTGGAGTTCAGAC-3′) primers. The PCR reaction was monitored using a CFX96 Real-Time PCR Detection System and stopped before the PCR reaction was fully saturated. The PCR product was purified with Agencourt AMPure XP beads.

For *S. coelicolor*, 1 μg of the total RNA instead of rRNA depleted RNA was ligated with 1 μL of 150 μM amino-blocked DNA adaptor (5′-p-NNAGATCGGAAGAGCGTCGTGT-3′) as described above. After ligation, rRNA was removed by using Hybridase™ Thermostable RNase H (Lucigen Corporation, Middleton, WI, USA). 13.5 μL of the purified ligation product was mixed with 1.5 μL of 10 × DNase I Reaction Buffer (New England BioLabs), 15 μL of Hybridase complement buffer (90 mM Tris-HCl pH 7.5, 200 mM KCl), 1 μL of anti-rRNA oligo mix (detailed composition is available in Figshare) and 2 μL of 50 mM MgCl_2_ and incubated in thermal cycler (heat to 95 °C, cool down to 65 °C)^30^. 2 μL of Hybridase™ Thermostable RNase H was added to the mixture and the mixture was incubated at 65 °C for 20 minutes, 90 °C for 1 second and 65 ^o^C for 10 minutes. rRNA depletion reaction was cleaned up using RNA Clean & Concentrator Kits (Zymo Research, Irvine, CA, USA) and the product was processed according to the remaining procedures as described above.

### High-throughput sequencing and data processing

All libraries were sequenced using either Illumina MiSeq or Illumina HiSeq. 2500 platform with either 1 × 100 bp (dRNA-Seq) or 1 × 50 bp (Term-Seq) read length except the dRNA-Seq of *S. tsukubaensis*. For the dRNA-Seq of *S. tsukubaensis*, both TAP(+) libraries and TAP(−) libraries were sequenced using Illumina MiSeq platform with 1 × 150 bp read length. The reads were processed using CLC Genomics Workbench. The raw reads were first mapped to phiX sequence, which is used in Illumina sequencing platform for quality control. The detailed mapping parameters are as follow. Mismatch cost: 2; Insertion cost: 3; Deletion cost: 3; Length fraction: 0.9; Similarity fraction: 0.9; Map randomly for non-specific matches. After mapping to phiX sequence, unmapped reads were collected and trimmed to remove adaptor sequences, short reads and low quality reads. The detailed parameters are as follow. Quality score limit: 0.05; Maximum number of ambiguities: 2; Remove adaptors; Discard read lengths below 15. For Term-Seq, two nucleotides at both ends were removed since the adaptors include random 2 nucleotides. The trimmed reads were mapped to the available reference genomes (Accession numbers: BA000030 for *S. avermitilis*, CP027858 and CP027859 for *S. clavuligerus*, NC_003888 for *S. coelicolor*, NC_010572 for *S. griseus*, CP009124 for *S. lividans*, CP020700 for *S. tsukubaensis*, CP059991 for *S. venezuelae*) with same parameters for phiX mapping, except the non-specific match handling (non-specific matches were discarded). After mapping to reference genomes, the directions of mapped reads of Term-Seq were inverted since the sequencing output comes in reverse direction.

### Identification of read count enriched positions

To determine the read count enriched peak positions where represent possible TSSs for dRNA-Seq or TTSs for Term-Seq, the read count enrichment to a specific position was represented with the z-score of the read count at the specific position as previously described^31^. The detailed calculation is as follow.

$$Z\left(x\right)=\frac{r\left(x\right)-m\left(C\left(x\right)\right)}{\sigma (C(x))}$$

*Z*(*x*) is the modified z-score at position *x*, *r*(*x*) is the read count of position *x*. The read counts were determined for 5′-ends of mapped reads for dRNA-Seq and 3′-ends of mapped reads for Term-Seq. *m*(*C*(*x*)) and *σ*(*C*(*x*)) are the mean and standard deviation of read counts of other positions, *C*(*x*), near the position *x*. *C*(*x*) is the set of positions y, satisfying 2 < |*x* − *y*| ≤ 50. The z-score of each position was first calculated separately for biological replicates and then averaged. Note that the position *x*, where *m*(*C*(*x*)) ≤ 0.25 in any of the biological replicates was ignored. For dRNA-Seq, the z-scores were calculated only for the positions of TAP(+) libraries, where normalized read counts are more than two-fold higher compared to the normalized read counts of the same positions in TAP(−) libraries. Finally, positions with z-scores higher than 12 were retained and if there are multiple positions within 3 nt distance, the positions with less z-scores were discarded.

## Data Records

For dRNA-Seq, raw read FASTQ files of three species (*S. avermitilis*, *S. clavuligerus*, *S. tsukubaensis*) were deposited in the National Center for Biotechnology Information (NCBI) Sequence Read Archive under the accession number SRP158023^32^, SRP188290^33^, and SRP103795^34^, respectively. The dRNA-Seq raw read FASTQ files of *S. griseus*, *S. lividans* and *S. venezuelae* were deposited in the European Nucleotide Archive (ENA) under the study accession number PRJEB40918^35^, PRJEB31507^36^ and PRJEB36379^37^, respectively. For Term-Seq of *S. avermitilis* and *S. lividans*, raw read FASTQ files were deposited under the same accession as the dRNA-Seq. For *S. clavuligerus*, the Term-Seq raw read FASTQ files were also deposited in the NCBI Sequence Read Archive under the accession number SRX6937123^38^ and SRX6937124^39^. For *S. coelicolor* and *S. griseus*, the Term-Seq raw read FASTQ files were deposited under the same accession as the dRNA-Seq of *S. griseus*. The Term-Seq raw read FASTQ files of *S. tsukubaensis* and *S. venezuelae* were deposited in the European Nucleotide Archive (ENA) under the study accession number PRJEB36379^37^.

The RNA-Seq data for the six *Streptomyces* species, *S. avermitilis*, *S. clavuligerus*, *S. coelicolor*, *S. lividans*, *S. tsukubaensis* and *S. venezuelae*, were retrieved from the accession number SRP158023^32^, SRP188290^33^, SRP058830^40^, PRJEB31507^36^, SRP103795^34^ and PRJEB34219^41^, respectively. And the RNA-Seq data for *S. griseus* were deposited under the same accession as the dRNA-Seq of *S. griseus* and Term-Seq of *S. coelicolor* and *S. griseus*. The predicted TSSs and TTSs along with the utilized python scripts were deposited in Figshare^30^. The brief summary of smBGC information and the detailed composition of anti-rRNA oligo mix utilized for depletion of rRNA of *S. coelicolor* were also uploaded in Figshare^30^.

## Technical Validation

### Evaluation of sequencing results

**Fig. 2:**
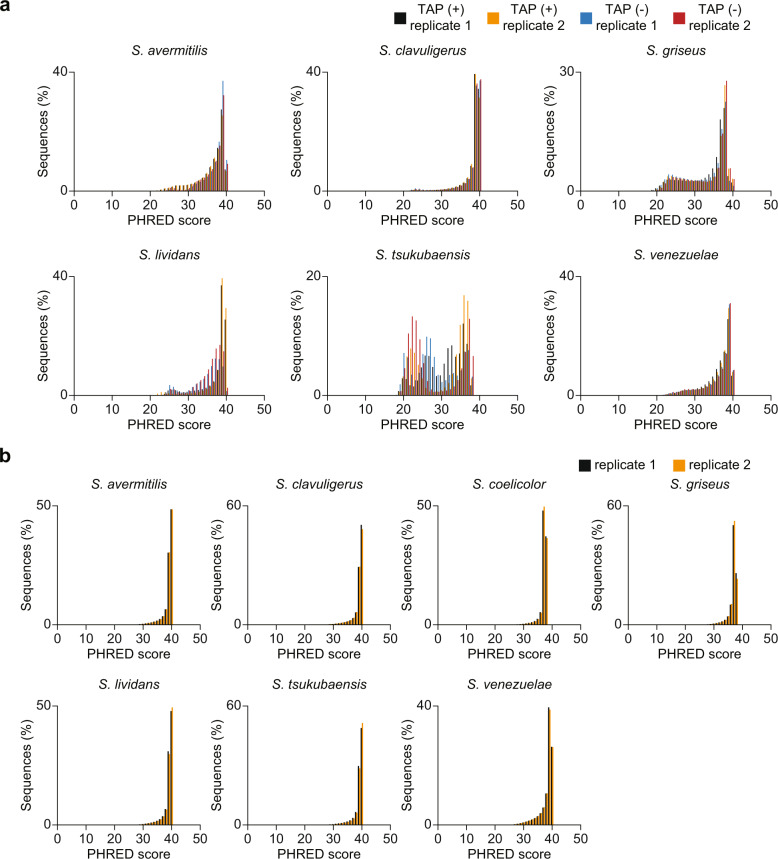
Technical Validation of dRNA-Seq and Term-Seq. (**a**) Average Phred score of dRNA-Seq reads after trimming. (**b**) Average Phred score of Term-Seq reads after trimming.

A total of 38 NGS libraries, including 24 dRNA-Seq libraries of *S. avermitilis*, *S. clavuligerus*, *S. griseus*, *S. lividans*, *S. tsukubaensis* and *S. venezuelae*, and 14 Term-Seq libraries of *S. avermitilis*, *S. clavuligerus*, *S. coelicolor*, *S. griseus*, *S. lividans*, *S. tsukubaensis* and *S. venezuelae* has been generated covering four different growth phases with biological replicates (dRNA-Seq data of *S. coelicolor* covering more diverse culture condition is available in the previous study performed by our group) (Fig. 1)^24^. The sequencing resulted in 4.97–26.60 and 3.47–16.1 million reads per library for dRNA-Seq and Term-Seq, respectively, after removing the phiX mapped reads (Tables 1 and 2). The retained reads were trimmed to remove adaptor sequences and discard short and low-quality reads. After trimming, the retained reads were subject to sequencing quality control, in terms of the Phred quality score^42^. Most reads showed average Phred quality score around 30–40, representing that the base-calling error probabilities in NGS runs are lower than 10^−3^ (Fig. 2a, b). After evaluating the quality of trimmed reads, the reads were mapped to the reference, resulting in 59.39% mapped reads for dRNA-Seq and 75.00% mapped reads for Term-Seq in average.

**Table 1:** Sequencing statistics of dRNA-Seq.

Species	Condition	Replicate	Raw reads	phiX unmapped reads	Total reads after trimming	Reads mapped to reference	Unmapped reads	Mapped reads %
*S. avermitilis*	TAP+	1	14,003,848	14,003,848	12,557,953	11,476,095	1,081,858	91.39%
		2	11,558,262	11,558,262	10,277,259	9,135,197	1,142,062	88.89%
	TAP−	1	8,677,978	8,677,978	8,237,481	7,867,315	370,166	95.51%
		2	7,009,201	7,009,201	5,962,196	5,314,941	647,255	89.14%
*S. clavuligerus*	TAP+	1	12,938,538	12,938,538	11,217,096	7,483,560	3,733,536	66.72%
		2	13,343,563	13,343,563	11,358,654	6,054,593	5,304,061	53.30%
	TAP−	1	12,951,898	12,951,898	10,578,113	4,607,972	5,970,141	43.56%
		2	13,489,136	13,489,136	11,424,071	4,859,586	6,564,485	42.54%
*S. griseus*	TAP+	1	25,211,934	24,970,901	6,099,314	745,696	5,353,618	12.23%
		2	26,886,707	26,604,864	8,121,887	1,168,272	6,953,615	14.38%
	TAP−	1	25,133,215	24,879,095	5,859,734	657,391	5,202,343	11.22%
		2	23,605,118	23,352,929	7,010,133	907,308	6,102,825	12.94%
*S. lividans*	TAP+	1	14,214,525	14,214,525	3,035,686	2,102,634	933,052	69.26%
		2	15,271,664	15,271,664	10,480,550	8,405,085	2,075,465	80.20%
	TAP−	1	8,317,597	8,317,597	2,883,147	2,033,201	849,946	70.52%
		2	7,287,887	7,287,887	4,518,829	3,358,219	1,160,610	74.32%
*S. tsukubaensis*	TAP+	1	6,922,568	6,729,100	6,397,214	3,629,503	2,767,711	56.74%
		2	5,221,139	4,971,846	4,740,361	2,626,013	2,114,348	55.40%
	TAP−	1	8,489,159	8,213,339	7,537,101	2,149,539	5,387,562	28.52%
		2	8,018,779	7,629,288	7,075,590	1,778,760	5,296,830	25.14%
*S. venezuelae*	TAP+	1	11,668,411	11,668,411	10,614,375	8,892,504	1,721,871	83.78%
		2	10,703,818	10,703,818	9,634,456	8,571,117	1,063,339	88.96%
	TAP−	1	12,519,347	12,519,347	10,983,952	9,293,311	1,690,641	84.61%
		2	13,326,417	13,326,417	11,740,020	10,115,801	1,624,219	86.17%

**Table 2:** Sequencing statistics of Term-Seq.

Species	Replicate	Raw reads	phiX unmapped reads	Total reads after trimming	Reads mapped to reference	Unmapped reads	Mapped reads %
*S. avermitilis*	1	12,838,857	12,838,857	12,351,833	9,619,400	2,732,433	77.88%
	2	12,149,121	12,149,121	11,653,771	7,766,714	3,887,057	66.65%
*S. clavuligerus*	1	10,019,122	10,019,122	9,837,011	7,958,067	1,878,944	80.90%
	2	9,107,580	9,107,580	8,253,492	5,466,897	2,786,595	66.24%
*S. coelicolor*	1	7,521,495	7,062,332	6,884,818	6,346,519	538,299	92.18%
	2	3,816,996	3,474,226	3,418,600	3,193,973	224,627	93.43%
*S. griseus*	1	6,402,323	6,359,534	5,987,607	3,630,800	2,356,807	60.64%
	2	5,689,021	5,645,990	5,367,423	3,441,188	1,926,235	64.11%
*S. lividans*	1	12,767,316	12,767,316	12,421,352	9,585,769	2,835,583	77.17%
	2	14,194,039	14,194,039	13,856,202	11,163,529	2,692,673	80.57%
*S. tsukubaensis*	1	16,091,519	16,091,519	13,639,245	10,165,845	3,473,400	74.53%
	2	13,220,796	13,220,796	12,256,943	10,161,126	2,095,817	82.90%
*S. venezuelae*	1	12,587,139	12,587,139	10,047,286	6,704,176	3,343,110	66.73%
	2	11,739,425	11,739,425	9,025,651	5,958,485	3,067,166	66.02%

### Assessment of sequencing datasets

**Fig. 3:**
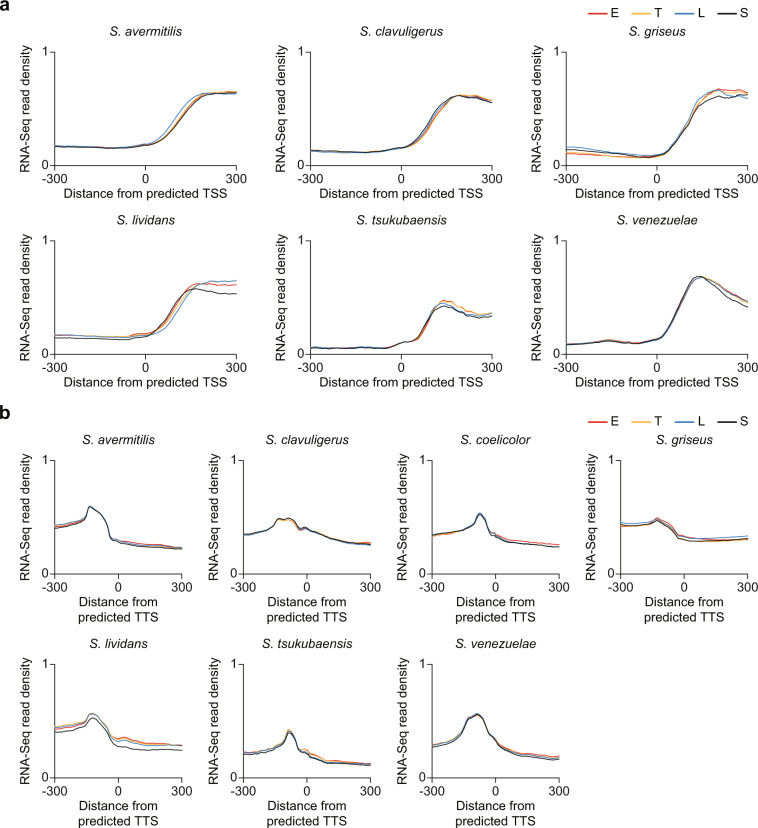
RNA-Seq read density across the predicted TSSs and TTSs. (**a**) RNA-Seq read density across the TSSs predicted from dRNA-Seq. (**b**) RNA-Seq read density across the TTSs predicted from Term-Seq. E, T, L and S represent the RNA-Seq read density of early-exponential, transition, late-exponential and stationary growth phases, respectively.

For technical validation of dRNA-Seq and Term-Seq results, the correspondence of read count enriched positions in dRNA-Seq and Term-Seq to increment and decrement of RNA-Seq profiles, respectively, was evaluated. The dRNA-Seq and Term-Seq read count enriched positions were determined for each *Streptomyces*, which highly likely represent the *in vivo* TSSs and TTSs, respectively (refer to Methods for detailed information about determination of read count enriched positions)^31^. In average, about 525 potential TSSs and 1285 potential TTSs were determined for each species, and about 7% of the predicted TSSs and 8% of the predicted TTSs were found in the smBGC regions (the smBGCs for each *Streptomyces* species were predicted using antiSMASH)^43^. The determined TSS and TTS information and smBGC information are available at Figshare^30^. Then, the RNA-Seq read density near the potential TSSs and TTSs was calculated (the RNA-Seq data were obtained from same culture conditions)^32–34,36,41^. Across the TSSs predicted from dRNA-Seq, RNA-Seq read density drastically increased for all the six *Streptomyces* species in four growth phases, indicating that the TSSs were successfully captured from dRNA-Seq (Fig. 3a). Likewise, RNA-Seq read density drastically decreased across the TTSs predicted from Term-Seq, indicating that the TTSs were successfully captured from Term-Seq for all the seven *Streptomyces* species (Fig. 3b).

## Data Availability

Read count enriched positions and the RNA-Seq read density across the positions were determined using two source codes in Python (version 3.5.2) programming language, which are publicly available in Figshare^30^.

## References

[1] Bérdy J (2005). Bioactive microbial metabolites. J Antibiot.

[2] Demain AL (1999). Pharmaceutically active secondary metabolites of microorganisms. Appl Microbiol Biotechnol.

[3] Demain AL (2006). From natural products discovery to commercialization: a success story. J Ind Microbiol Biotechnol.

[4] Silver LL (2011). Challenges of antibacterial discovery. Clin Microbiol Rev.

[5] Bentley SD (2002). Complete genome sequence of the model actinomycete *Streptomyces coelicolor* A3(2). Nature.

[6] Nett M, Ikeda H, Moore BS (2009). Genomic basis for natural product biosynthetic diversity in the actinomycetes. Nat Prod Rep.

[7] Ventola CL (2015). The antibiotic resistance crisis: part 1: causes and threats. P T.

[8] Challis GL, Hopwood DA (2003). Synergy and contingency as driving forces for the evolution of multiple secondary metabolite production by *Streptomyces* species. Proc Natl Acad Sci USA.

[9] Bibb MJ (2005). Regulation of secondary metabolism in streptomycetes. Curr Opin Microbiol.

[10] Bursy J (2008). Synthesis and uptake of the compatible solutes ectoine and 5-hydroxyectoine by *Streptomyces coelicolor* A3(2) in response to salt and heat stresses. Appl Environ Microbiol.

[11] Lee, N. *et al*. Iron competition triggers antibiotic biosynthesis in Streptomyces coelicolor during coculture with Myxococcus xanthus. *ISME J*, 10.1038/s41396-020-0594-6 (2020). 10.1038/s41396-020-0594-6 PMC7174319 31992858

[12] Bervoets I, Charlier D (2019). Diversity, versatility and complexity of bacterial gene regulation mechanisms: opportunities and drawbacks for applications in synthetic biology. FEMS Microbiol Rev.

[13] Browning DF, Busby SJ (2004). The regulation of bacterial transcription initiation. Nat Rev Microbiol.

[14] Cho BK (2009). The transcription unit architecture of the *Escherichia coli* genome. Nat Biotechnol.

[15] Dar D (2016). Term-seq reveals abundant ribo-regulation of antibiotics resistance in bacteria. Science.

[16] Hwang S (2019). Primary transcriptome and translatome analysis determines transcriptional and translational regulatory elements encoded in the *Streptomyces clavuligerus* genome. Nucleic Acids Res.

[17] Lee Y (2019). The Transcription Unit Architecture of *Streptomyces lividans* TK24. Front Microbiol.

[18] Burg RW (1979). Avermectins, new family of potent anthelmintic agents: producing organism and fermentation. Antimicrob Agents Chemother.

[19] Paradkar A (2013). Clavulanic acid production by *Streptomyces clavuligerus*: biogenesis, regulation and strain improvement. J Antibiot (Tokyo).

[20] Barreiro C (2012). Draft genome of *Streptomyces tsukubaensis* NRRL 18488, the producer of the clinically important immunosuppressant tacrolimus (FK506). J Bacteriol.

[21] Waksman SA (1953). Streptomycin: background, isolation, properties, and utilization. Science.

[22] Myronovskyi M, Luzhetskyy A (2019). Heterologous production of small molecules in the optimized *Streptomyces* hosts. Nat Prod Rep.

[23] Anné J, Vrancken K, Van Mellaert L (2014). Van Impe, J. & Bernaerts, K. Protein secretion biotechnology in Gram-positive bacteria with special emphasis on *Streptomyces lividans*. Biochim Biophys Acta.

[24] Jeong Y (2016). The dynamic transcriptional and translational landscape of the model antibiotic producer *Streptomyces coelicolor* A3(2). Nat Commun.

[25] Dar D, Sorek R (2018). High-resolution RNA 3′-ends mapping of bacterial Rho-dependent transcripts. Nucleic Acids Res.

[26] Bailey TL (2009). MEME SUITE: tools for motif discovery and searching. Nucleic Acids Res.

[27] Shine J, Dalgarno L (1974). The 3′-terminal sequence of *Escherichia coli* 16S ribosomal RNA: complementarity to nonsense triplets and ribosome binding sites. Proc Natl Acad Sci USA.

[28] Morris DR, Geballe AP (2000). Upstream open reading frames as regulators of mRNA translation. Mol Cell Biol.

[29] Garst, A. D., Edwards, A. L. & Batey, R. T. Riboswitches: structures and mechanisms. *Cold Spring Harb Perspect Biol* **3**, 10.1101/cshperspect.a003533 (2011). 10.1101/cshperspect.a003533 PMC3098680 20943759

[30] Lee, Y. *et al*. Genome-scale determination of 5′ and 3′ boundaries of RNA transcripts in *Streptomyces* genomes. *figshare*10.6084/m9.figshare.c.5044730 (2020). 10.1038/s41597-020-00775-w PMC7738537 33319794

[31] Lalanne JB (2018). Evolutionary Convergence of Pathway-Specific Enzyme Expression Stoichiometry. Cell.

[32] (2020). NCBI Sequence Read Archive.

[33] (2019). NCBI Sequence Read Archive.

[34] (2019). NCBI Sequence Read Archive.

[35] (2020). European Nucleotide Archive.

[36] (2019). European Nucleotide Archive.

[37] (2020). European Nucleotide Archive.

[38] (2020). NCBI Sequence Read Archive.

[39] (2020). NCBI Sequence Read Archive.

[40] (2016). NCBI Sequence Read Archive.

[41] (2019). European Nucleotide Archive.

[42] Ewing B, Hillier L, Wendl MC, Green P (1998). Base-calling of automated sequencer traces using phred. I. Accuracy assessment. Genome Res.

[43] Blin K (2019). antiSMASH 5.0: updates to the secondary metabolite genome mining pipeline. Nucleic Acids Res.

